# MRI and ultrasound versus conventional measures of disease activity and structural damage in evaluating treatment efficacy in JIA. Do these imaging techniques have an additional value?

**DOI:** 10.1186/1546-0096-9-S1-O12

**Published:** 2011-09-14

**Authors:** C Malattia, A Consolaro, S Pederzoli, MB Damasio, R Hasija, M Mazzoni, A Madeo, A Beltramo, S Viola, A Buoncompagni, C Mattiuz, A Ravelli, A Martini

**Affiliations:** 1 Pediatria II, IRCCS G. Gaslini, Genova, Italy; 2 UO Radiologia, IRCCS G. Gaslini, Genova, Italy; 3 Department of Pediatrics, University of Genova, Italy

## Background

In recent years, MRI and US have been increasingly used as outcome measures in clinical trials in rheumatoid arthritis; their value in evaluating treatment efficacy has never been tested in juvenile idiopathic arthritis (JIA).

## Aim

To compare conventional measures with MRI and US in the assessment of treatment efficacy in JIA.

## Methods

Patients with JIA and active wrist arthritis who started receiving a second line therapy (DMARDs or biological agent) at the study Unit between 2008 and 2010 were enrolled in the study. The clinically more affected wrist was studied with MRI (1.5 T), US and conventional radiography (CR), coupled with standard clinical assessment at the study enrollment and after 1 year. Patients were characterized according to the pACR response criteria, and to the current criteria for inactive disease.

## Results

Thirty seven patients (median age 10.8 years, median disease duration 4.1 years) were enrolled. Thirteen patients out of 37 patients (35.1%) started DMARDs while 24/37 patients (64.8%) started biologic agents. Patients who met the definition of improvement according to the pACR90 criteria (N=13) showed a significantly greater decrease in MRI synovitis score compared to patients who met the pACR 30, 50 and 70 criteria (N=14) (p<0.0023), and compared to the non-responders (N=10) (p<0.002), indicating the ability of MRI synovitis score to discriminate between different levels of responder categories. Excluding physician's global assessment of disease activity, MRI synovitis score was more responsive (SRM 1.61) than ACR core set measures and US synovitis score (SRM gray scale 0.87; SRM Doppler 0.71). After 1 year 11/37 patients met the critera for inactive disease. Of these, only 1 showed no synovitis according to MRI and 4 according to US. MRI and CR performed equally in detecting structural damage progression after 1 year.

## Conclusions

The excellent responsiveness and discriminant ability of MRI synovitis score makes it promising as an outcome measure in clinical studies. Only the high degree responders showed a significant decrease in synovitis score. Clinical criteria are insensitive to detect low level of inflammation accurately, suggesting the potential role of imaging in the assessment of remission.

